# Two new species of *Thyridosmylus* Krüger, 1913 from Madagascar (Neuroptera, Osmylidae)

**DOI:** 10.3897/zookeys.724.21057

**Published:** 2017-12-21

**Authors:** Han Xu, Shaun L. Winterton, Yongjie Wang, Zhiqi Liu

**Affiliations:** 1 Department of Entomology, China Agricultural University, Beijing 100094, China; 2 California State Collection of Arthropods, California Department of Food & Agriculture, Sacramento, California, USA; 3 College of Life Sciences, Capital Normal University, Beijing 100048, China

**Keywords:** lacewing, Malagasy species, Osmylidae

## Abstract

The lance lacewing genus *Thyridosmylus* Krüger (Osmylidae: Spilosmylinae) is found in Madagascar and Southeast Asia. Two new Malagasy species are described herein, *Thyridosmylus fuscomarginatus* Xu, Wang & Winterton, **sp. n.**, and *Thyridosmylus longiprocessus* Xu, Wang & Winterton, **sp. n.** A key to differentiate the Malagasy species of *Thyridosmylus* is provided.

## Introduction

*Thyridosmylus* Krüger is a small genus of lance lacewings assigned to the subfamily Spilosmylinae. Krüger (1913) established the genus based on the species *Osmylus langii* McLachlan, 1870, characterized by distinctively marked forewings commonly with fenestrate spots around the outer gradate cross-veins. In 1917, Navás erected the genus *Centrolysmus* based on *Osmylus perspicillaris*, but in the subsequent revision of *Thyridosmylus* Kimmins (1942) suggested that *O. perspicillaris* (and subspecies) should be instead placed in *Thyridosmylus*. Unfortunately, Kimmins mistook *Centrolysmus epiphanes* as the type species of *Centrolysmus* and thus inadvertently maintained the validity of *Centrolysmus*. Oswald and Penny (1991) clarified this mistake and formally synonymized *Centrolysmus* with *Thyridosmylus*.

Presently there are 19 species described in *Thyridosmylus*, including 17 species from South East Asia and two from Madagascar, suggesting the conspicuously disjunct geographical distribution (Gerstaecker 1884; Navás 1933; Kimmins 1942; Fraser 1955; Yang 1987, 1988, 1992, 1993, 1999, 2002; Yang et al. 1995; Wang et al. 2008, 2011). The congeneric status of all species in the genus has been routinely accepted by subsequent authors (Wang et al. 2008, 2011; Winterton et al. 2017); in a cladistics analysis of *Thyridosmylus*, Wang et al. (2011) concluded the Malagasy species occupy a sister position to the Oriental species, suggesting the southern origin of this genus should be no later than Late Cretaceous. Indeed, in their phylogenetic analyses of Osmylidae, Winterton et al. (2017) deduced that the divergence of *Thyridosmylus* from its sister genus *Spilosmylus* Kolbe occurred during the Middle Jurassic (177 Mya), and that early splits in both genera were caused by vicariance resulting from the subsequent rafting of India.

The Malagasy *Thyridosmylus* species have been mentioned seldom in the literature since their description. Based on recently collected material from Madagascar, two new species are described, *Thyridosmylus fuscomarginatus* Xu, Wang & Winterton, sp. n. and *Thyridosmylus longiprocessus* Xu, Wang & Winterton, sp. n.

## Materials and methods

The specimens observed in this study are deposited in the California Academy of Science, San Francisco (CASC), California State Collection of Arthropods, Sacramento (CSCA) and Entomological Museum of China Agricultural University, Beijing (CAU). Terminalia preparations were made by macerating the apex of the abdomen in hot 10% KOH for 3–5 min, neutralized with 10% acetic acid. The apex of the abdomen was then transferred to glycerol for further dissection and examination. After examination, they were moved to fresh glycerol and stored in a microvial pinned below the specimens. Image of wings were taken with a Nikon D7000 digital camera. Drawings were made under a light microscope. The terminology for wing venation and genitalia follows Winterton and Wang (2016) and Winterton et al. (2017).

## Taxonomy

### Genus Thyridosmylus

Taxon classificationAnimaliaNeuropteraOsmylidae

Krüger

Thyridosmylus Krüger, 1913: 87. Type species: Osmylus langii McLachlan, 1870: 197. Original designation. Type locality: Masuri (India).
Centrolysmus Navás, 1917: 15. Type species: Osmylus perspicillaris Gerstaecker, 1884: 46. Original designation. Type locality: Darjeeling (India).

#### Diagnosis.

Medium sized species (forewing length 13–24 mm), wings narrow (maximum width of forewing 4–7 mm). Ocelli present. Forewing strongly patterned with distinctive fenestrate spots close to the outer gradate series, with numerous suffusions, some of these forming a large macula, numerous crossveins. Costal crossveins simple, subcostal space with single crossvein. At least two distinct gradate series. Area between M and Cu lacking crossveins between them basally, thus a large cell is present. Hindwing with Cu forked at base, CuP short and not pectinate. Male ectoproct bearing a dorsal digitiform projection, gonarcus symmetrical, amalgamated distally, sclerotized marginally; baculum present; mediuncus C-shaped, linked by membranes; parameres present. Female genitalia with sternite 8 small, gonapophyses 9 as paired sclerites closely associated with gonocoxites 9, spermatheca simple or with a complicated lobed morphology.

#### Redescription.

Body length 10–15 mm. Head brown or dark brown; antennae yellow and shorter than or equal to half of length of forewing, scape and pedicel dark brown, flagellum yellow; compound eyes black; ocellar tubercles yellowish to brown; labrum brown or dark brown. Thorax dark brown with long setae; meso- and metathorax dark brown. Legs yellow with brown setae. Forewing length 13–24 mm, width 4–7 mm. Forewing generally with characteristic fenestrate spots near the outer gradate series and with numerous fuscous markings, membrane hyaline or with light infuscate suffusion; pterostigma brown with a light brown centre; two nygmata as brown spots; venation brown, some cross-veins edged with brown markings; costal cross-veins simple and occasionally bifurcate; cross-vein sc-r1 close to the base of forewing; forewing Rs with 10–15 branches, cross-veins among Rs branches forming more than two series of gradates; basal mp-cu cross-vein only one, forming a large cell. Hindwing length 12–22 mm, width 4–7 mm. Hindwing generally hyaline with few spots; pterostigma light yellow; nygmata inconspicuous and light brown; base of MP with a spur. Male genitalia. Tergite 8 and sternite 8 approximately quadrate. Tergite 9 commonly narrow, sternite 9 approximately trapezoidal or triangular; ectoproct with a dorsal process, callus cercus round; gonarcus sclerotized marginally, symmetrical and fused distally and base connected with a goblet-shaped anterior apodeme; entoprocesses bent in middle; mediuncus lobes bent into C-shape laterally and fused at base; parameres arch-like with medial thickening and invariant within genus. Female genitalia. tergite 8 broad and approximately quadrate; sternite 8 reduced and small; tergite 9 narrow and commonly constricted in middle articulated with gonopophysis 9 + gonocoxite 9; gonocoxite 9 finger-like in lateral view; each spermatheca connected with a spermathecal duct.

#### Comments.

The distinction between the closely related *Thyridosmylus* and *Spilosmylus* has been historically ambiguous, although their reciprocally monophyletic sister-group relationship was confirmed in the phylogeny of the family by Winterton et al. (2017). Traditionally, *Thyridosmylus* was characterized by numerous markings of forewings with the often presence of distinct fenestrations around the outer gradate cross veins, while the forewings of *Spilosmylus* are characterised by having fewer maculations and an embossed spot along the posterior wing margin, or intermittent dark streaks between Sc and R_1_. As more species have been described it seems that these characters could not completely separate these genera, with numerous instances where the diagnostic feature of either genus is lacking. With regard to *Thyridosmylus*, fenestrate markings are not found in several species such as *T. fuscus* Yang, 1999, *T. longiprocessus* Xu, Wang & Winterton, sp. n., *T. maolanus* Yang, 1993, *T. marmoratus* Fraser, 1955, *T. pallidius* Yang, 2002 and *T. trifasciatus* Yang, 1993. Still, the forewings of these species exhibit extensive fragmental markings and do not possess embossed spots and intermittent streaks typical among *Spilosmylus* species. Additionally, the spermathecae in some *Thyridosmylus* species (e.g., *T. langii*, *T. paralangii* and *T. fuscomarginatus* Xu, Wang & Winterton, sp. n.) are complex and multilobed, resembling those of some *Spilosmylus* species. The shape of the external genitalia in the males of *Thyridosmylus* varies less compared with *Spilosmylus*.

The forewing of *Thyridosmylus* has two m-cu cross veins between the stem of the medial vein (before the split into MA and MP) and cubital vein to form single large cell, while the forewing of *Thaumatosmylus* has few fuscous spots, and three m-cu cross veins to form two basal cells. Also, the spermathecae in females of *Thyridosmylus* are elliptical or multilobed while the spermathecae in *Thaumatosmylus* tend to be large with a basal club-like sac, or with a small and apical finger-like to ovoid sac. Although the definition of genus *Glenosmylus* has been obscure since it was erected by Krüger (1913), it can be distinguished from *Thyridosmylus* based on three m-cu cross veins in the forewing character, similar to that found in *Thaumatosmylus*.

#### Key to *Thyridosmylus* species in Madagascar

**Table:** 

1	Outer margin of forewing with extensive fuscous markings with a distinct fenestration around the outer gradate cross veins (Fig. 1)	***Thyridosmylus fuscomarginatus* Xu, Wang & Winterton, sp. n.**
–	Outer margin of forewing extensively marked but lacking distinct fenestration near outer gradates	**2**
2	Hindwing with series of small dark spots along the posterior margin towards the wing apex	***Thyridosmylus punctulatus* (Navás, 1933)**
–	Hindwing without series of spots along posterior margin towards the wing apex (Fig. 3)	**3**
3	Basal half of forewing with two brown bands (Fig. 3)	***Thyridosmylus longiprocessus* Xu, Wang & Winterton, sp. n.**
–	Forewing without brown band-like markings	***Thyridosmylus marmoratus* Fraser, 1955**

### Thyridosmylus fuscomarginatus

Taxon classificationAnimaliaNeuropteraOsmylidae

Xu, Wang & Winterton sp. n.

http://zoobank.org/D9B51BB6-96F2-4285-A62C-1A291EF370F7

Figs 1 , 2

#### Diagnosis.

Pronotum light yellow with three longitudinal dark brown stripes; posterior margin of forewing with brown markings, branches of vein A_1_ marked with a dark brown spot, area around outer gradate cross-veins fenestrate with pale venation; sternite 8 in female reduced, extending anteriorly to form a forward process in lateral view; spermatheca complex with 11–12 sacs, basal sac large.

**Figure 1.**
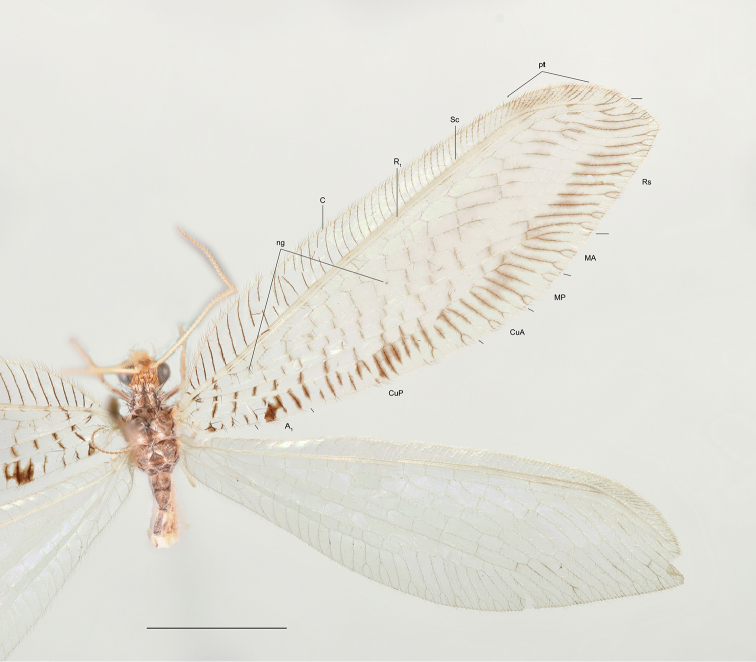
Wings of *Thyridosmylus fuscomarginatus* Xu, Wang & Winterton, sp. n. Abbreviations: ng, nygmata; pt, pterostigma; og, outer gradates. Scale bar: 0.5 mm.

#### Description.

**Figure 2.**
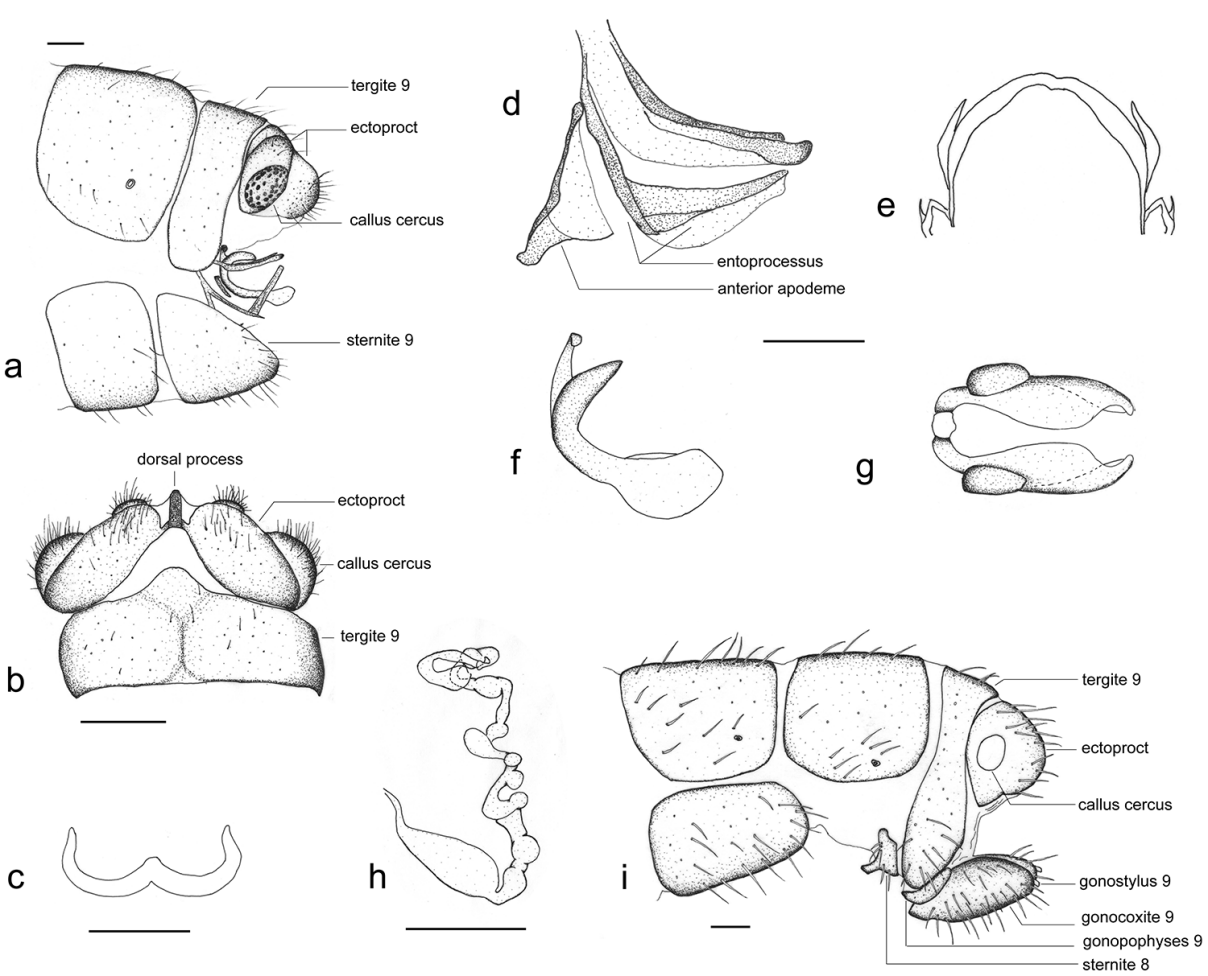
*Thyridosmylus fuscomarginatus* Xu, Wang & Winterton, sp. n. Male : **a, b** abdomen terminalia, lateral view (**a**) and dorsal view (**b**) **c** parameres, dorsal view **d–e** gonarcus, lateral view (**d**) and dorsal view (**e**) **f ,g** mediuncus, lateral view (**f**) and dorsal view (**g**). Female: **h** spermatheca **i** abdomen terminalia, lateral view. Scale bars: 0.2 mm.

*Head.* Vertex yellowish-brown with black setae; compound eyes black, ocelli yellow, each one anteriorly edged with a black spot. Antennal flagellum light yellow with dark yellow apex; scape and pedicel light yellow; frons brown. *Thorax.* Pronotum light yellow with a dark brown longitudinal stripe in middle and parallel two dark brown markings on both sides, three markings linked by a latitudinal light brown stripe; meso- and metanotum brown. *Legs.* Yellow with brown setae; claws brown. *Wings* (Fig. 1). Forewing length 17–18 mm, width 5–6 mm; membrane hyaline; cross-veins in the basal half of costal field dark brown, paler distally; pterostigma light brown; Rs with 13–14 branches; distal part of branches of vein Rs, M and Cu edged with brown markings, branches of A_1_ edged with an irregular and clear dark brown spot; area around outer gradate cross-veins hyaline with pale venation, appearing fenestrate; cross-veins among branches of Rs, M, Cu and A edged with fuscous markings. Hindwing length 15–16 mm, width 4–5 mm; membrane hyaline; veins yellowish; pterostigma light brown; Rs with 11 branches, cross-veins among Rs branches forming two series of gradates; basal MP with a spur. *Male genitalia* (Fig. 2a–g). Tergite 8 and sternite 8 quadrangular with brown setae; tergite 9 narrow and extended distally in dorsal view; sternite 9 subtriangular in lateral view; ectoproct with a dorsal rod-like process (Fig. 2b), base inflated and distal part with numerous brown setae; callus cercus approximately rounded; gonarcus approximately triangular in lateral view (Fig. 2d) and narrow arch-like in dorsal view (Fig. 2e), basally articulated internally with tergite 9, gonarcus dorsally sclerotized and ventrally membranous (Fig. 2d); entoprocessus narrow and reflexed dorsally with a backward process at the corner of bend, distal margin membranous (Fig. 2d); gonarcus anterior apodeme goblet-shaped; mediuncus lobes C-shaped in lateral view (Fig. 2f), thickened basally and sclerotized well but distal part translucent and expanding dorsal-medially and ventrally (Fig. 2g); parameres sclerotized, horn-shaped, and thickened medially (Fig. 2c). *Female genitalia* (Fig. 2h–i). Tergite 8 quadrate, sternite 8 reduced, close to tergite 9 and extending anteriorly to form a forward process in lateral view; tergite 9 narrow and constricted at the level of ectoproct; gonopophyses 9 and gonocoxite 9 closely associated, gonocoxite 9 thick finger-like with gonostylus 9 distally; ectoproct coniform, callus cercus rounded; spermathecae complex, each comprised of 11–12 sacs, basal sac large and long (Fig. 2h).

#### Material examined.

**Holotype.** Female. MADAGASCAR: Mahajanga Prov., Parc National de Baie de Raly, 12.4 km, 337° NNW Soalala, elev. 10 m, 16°00'36"S, 45°15'54"E, 26–30.xi.2002, coll. Fisher, Griswold et al., collected at night, tropical dry forest (CASC). **Paratypes.** 1 male. MADAGASCAR: Mahajanga Prov., Forêt de Tsimembo, 8.7 km, 336° NHW Soatana, elev. 20 m, 19°1'17"S, 44°26'26"E, 21–25.xi.2001, coll. Fisher, Griswold et al., collected at night, tropical dry forest (CASC). 1 female. MADAGASCAR: Mahajanga Prov., Parc National de Baie de Raly, 12.4 km, 337° NNW Soalala, elev. 10 m, 16°00'36"S, 45°15'54"E, 26–30.xi.2002, coll. Fisher, Griswold et al. Collected at night, tropical dry forest (CASC). 1 female. MADAGASCAR: Antsiranana Prov., Ankarana National Park, Aurelien Hotel, 140 m, 12°58’07.5”S 49°08’12.8”E, 16.xii.2016, coll. Hu Li. (CAU). 1 male. MADAGASCAR: Antsiranana Prov., 3 km, W Sakalava Beach, 12°17'10"S 49°22'00"E, 40 m, 21–23.i.2001, coll. M. E. Irwin, E. I. Schilinger, R. Harin’ Hala. (CASC).

#### Etymology.

The specific name “*fuscomarginatus*”, a compound from Latin *fusco*- (fuscus) and *marginatus*- (margin), in reference to the colour and pattern of markings on the outer and posterior margin of forewings.

#### Distribution.

Madagascar (Antsiranana, Mahajanga)

#### Remarks.

The forewings markings of *Thyridosmylus fuscomarginatus* sp. n. are characteristic, clearly differed from other *Thyridosmylus* species by the dark posteromarginal stripe.

### Thyridosmylus longiprocessus

Taxon classificationAnimaliaNeuropteraOsmylidae

Xu, Wang & Winterton sp. n.

http://zoobank.org/BA10CB95-0E12-4E12-AF4F-4699EC2F838F

Figs 3 , 4

#### Diagnosis.

Frons brown with two dark brown markings; forewing hyaline, basal half with two brown stripes; male genitalia with ectoproct bearing a long dorsal rod-like process; distal part of gonarcus with abundant pilosity; basal part of mediuncus laterally with heart-shaped structures in dorsal view; spermatheca complex, with 13 sacs, basal one small and oval.

**Figure 3.**
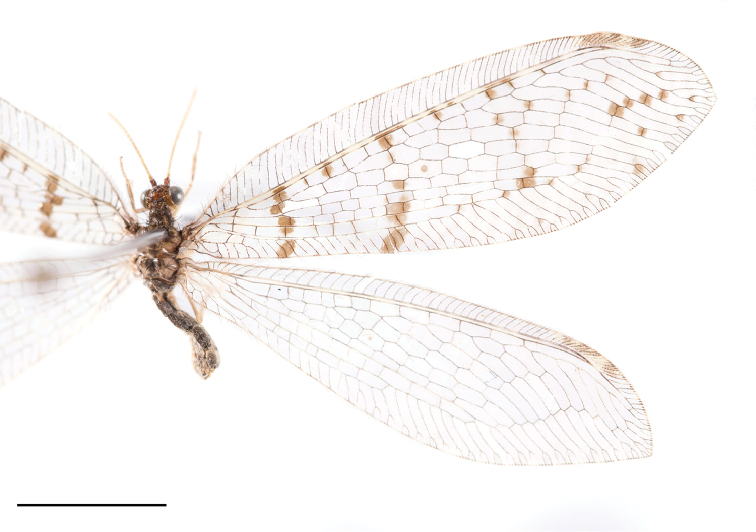
Wings of *Thyridosmylus longiprocessus* Xu, Wang & Winterton, sp. n. Scale bar: 0.5 mm.

#### Description.

**Figure 4.**
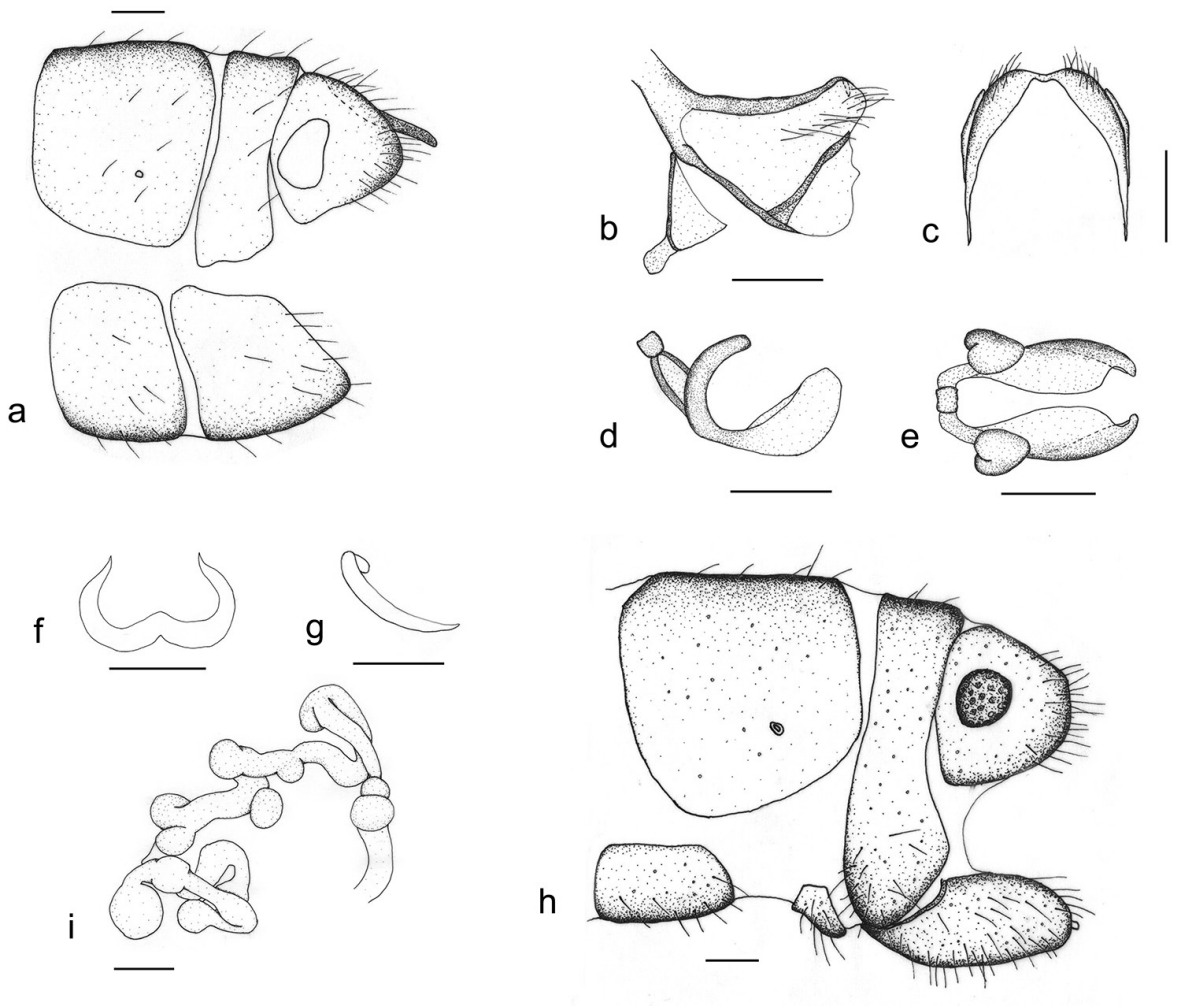
*Thyridosmylus longiprocessus* Xu, Wang & Winterton, sp. n. Male : **a** abdomen terminalia, lateral view; **b, c** gonarcus, lateral view (**b**) and dorsal view (**c**) **d, e** mediuncus, lateral view (**d**) and dorsal view (**e**) **f, g** parameres, dorsal view (**f**) and lateral view (**g**). Female: **h** abdomen terminalia, lateral view **i** spermatheca. Scale bars: 0.2 mm.

*Head.* Vertex brown with black setae; compound eyes grey, ocelli yellow, base edged with a dark brown spot. Antennal flagellum yellow; scape and pedicel brown; frons brown with two dark brown markings. *Thorax.* Pronotum black; meso- and metanotum brown with a longitudinal dark stripe in middle. *Legs.* Legs yellow with brown setae; claws brown. *Wings* (Fig. 3). Forewing length 17–18 mm, width 5–6 mm; membrane hyaline, basal half with 10 brown spots forming two stripes; veins brown; pterostigma light brown; Rs with 12 branches, cross-veins among Rs branches forming two series of gradates, outer gradate cross-veins edged with brown spots; forewing M branching more basally than the divergence of basal branch of Rs; nygmata clear, distal nygma edged with a rounded light brown spot. Hindwing length 15–16 mm, width 4–5 mm; membrane hyaline; veins brown; pterostigma light brown. Rs with 12 branches, cross-veins among Rs branches forming two series of gradates. Basal MP with a spur. *Male genitalia* (Fig. 4a–g). Tergite 8 and sternite 8 quadrangular with brown setae; tergite 9 narrow; sternite 9 quadrangular; ectoproct with a long dorsal rod-like process (Fig. 4a); callus cercus approximately oval; gonarcus approximately triangular in lateral view and narrow and arched in dorsal view; gonarcus membranous posterolaterally with dense setal pile, dorsal margin well sclerotized (Fig. 4b–c); entoprocessus narrow, bent dorsad, with a posteroventral pointed projection, distal region membranous (Fig. 4b); mediuncus lobes C-shaped in lateral view (Fig. 4d), basally thickened and well sclerotized, distal part translucent and expanding dorsal-medially and ventrally; basal part laterally with heart-shaped structures in dorsal view (Fig. 4e); parameres sclerotized, horn-shaped in dorsal view, thickened medially and bent in lateral view (Fig. 4f–g). *Female genitalia* (Fig. 4h–i). Tergite 8 quadrate, sternite 8 reduced, close to tergite 9; tergite 9 narrow and constricted at the level of ventral margin of ectoproct; gonopophyses 9 and gonocoxite 9 closely associated, gonocoxite 9 finger-like with gonostylus 9 distally; ectoproct coniform, callus cercus rounded; spermathecae complex, each comprised of 13 sacs, basal sac small and oval (Fig. 4i).

#### Material examined.

**Holotype.** Male. MADAGASCAR: Fianarantsoa Prov., S. E. Fandriana Korikory, elev. 1670 m, 20°23'S, 47°40'E, 13.iii.2002, coll. Michael E. Irwin & Evert I. Schlinger (CASC). **Paratype**. Female. Data same as holotype.

#### Etymology.

Thve specific name “*longiprocessus*”, a compound from Latin *longi*- (long) and *processus*- (process), which refers to the long dorsal process of ectoproct in male.

#### Distribution.

Madagascar (Fianarantsoa).

#### Remarks.

The male genitalia are not well sclerotized probably because it was teneral when it was collected. The sternite 8 in *T. longiprocessus* sp. n. is reduced into a sclerite without processes and the spermatheca is complex, consisting of 13 sacs, of which, the basal one is small and oval. Moreover, the dorsal process of ectoproct in male is quite long, clearly distinguished from other *Thyridosmylus* species, in which it is inconspicuous when it is observed in lateral view.

## Supplementary Material

XML Treatment for Thyridosmylus

XML Treatment for Thyridosmylus fuscomarginatus

XML Treatment for Thyridosmylus longiprocessus
